# Mapping between EQ‐5D‐3L and EQ‐5D‐5L: A survey experiment on the validity of multi‐instrument data

**DOI:** 10.1002/hec.4487

**Published:** 2022-02-28

**Authors:** Mónica Hernández‐Alava, Stephen Pudney

**Affiliations:** ^1^ School of Health and Related Research University of Sheffield Sheffield UK

**Keywords:** EQ‐5D, mapping, randomised experiment, survey reporting, UKHLS

## Abstract

EQ‐5D is a 5‐item questionnaire instrument designed to measure health‐related quality of life. It is extremely important, since it is used to measure health benefits in many studies providing evidence for reimbursement decisions by the National Institute for Health and Care Excellence in England and similar policy bodies in other countries. EQ‐5D has been redesigned in a more detailed form (EQ‐5D‐5L), but much existing cost‐effectiveness evidence is based on the older version (EQ‐5D‐3L). Statistical mapping from one version to another is widely used, exploiting data from multi‐instrument surveys incorporating both variants. However, little is known about the robustness of data from such multi‐instrument surveys. We design a randomized experiment to investigate whether inclusion of both versions at different stages in a single interview gives a reliable picture of the relationship between health measures from the two instruments and embed it in individual interviews from the UK Understanding Society household panel. We find that sequencing of the two versions of EQ‐5D within an interview has a significant impact not only on the resulting data but also on the estimated mapping models. We illustrate the non‐negligible effects in two real‐world cost‐effectiveness examples and discuss the implications for future multi‐instrument survey design.

## 1 INTRODUCTION

EQ‐5D is a 5‐item questionnaire module designed to measure health‐related quality of life across five domains or dimensions: mobility, self‐care, usual activities, pain/discomfort and anxiety/depression. The original EQ‐5D, now known as EQ‐5D‐3L (shortened to 3L henceforth), allows individuals to describe their degree of health impairment using three levels of severity (no problems/some problems/extreme problems). Altogether, 243 different health states can be described in this way. Dolan (1997) estimated “utility weights” or values for each one of the 243 health states using the preferences of a sample of the general population for the UK. The resulting set of values has an upper bound at one representing full health with zero being equivalent to being dead. Values below zero denote health states that are considered to be worse than being dead. Although simple, EQ‐5D has become extremely important for policy purposes. Economic evaluations providing evidence for decisions on medical technologies designed to improve health such as those used by the National Institute for Health and Care Excellence (NICE) in England and similar policy bodies in other countries use the quality‐adjusted life year (QALY) as their measure of benefit. The QALY reflects concerns for both the quality and length of life and it is based on patient reported outcome measures such as EQ‐5D, the preferred health benefit measure of NICE for its technology appraisals (NICE, 2013).

EQ‐5D has been redesigned (Herdman et al., 2011) in a more detailed form known as EQ‐5D‐5L (shortened to 5L henceforth), adding two more levels in between levels 1 and 2, and 2 and 3 of the original 3L version. Recent work by the NICE Decision Support Unit (Hernández‐Alava, Pudney, & Wailoo, 2018; Pennington et al., 2018) has revealed substantial differences between the way in which 3L and 5L estimate gains for health technologies in terms of Quality Adjusted Life Years (QALYs) and, in turn, their cost‐effectiveness. These differences originate in the responses individuals give to the two descriptive systems and also the valuations assigned to health states by the available utility scoring systems. These measurement conflicts create a real policy dilemma facing health authorities in recommending one or other of the two versions of EQ‐5D. Whichever is recommended, there exists a stock of existing evidence generated in 3L terms and a growing body of 5L‐based evidence that will need to be reconciled or combined in decision models for many years to come: a 5L value set will not make 3L evidence redundant. It is highly likely that there will also be new extensions and variants of measures developed in the future for which the same issues will apply. Statistical mapping from one version to another is widely used to negotiate these obstacles, exploiting multi‐instrument (MI) surveys which incorporate both variants (see Hernández‐Alava & Pudney, 2017, Pennington et al., 2018, van Hout & Shaw, 2021, van Hout et al., 2012, and Wailoo et al., 2017 for examples of mapping between 3L and 5L).^1^

Two existing datasets have been identified previously for mapping between the two versions of EQ‐5D but both have limitations in terms of sample size and study design for statistical mapping (Hernández‐Alava et al., 2017). FORWARD, or the National Databank for Rheumatic Diseases (Wolfe & Michaud, 2011), is a register of patients with rheumatoid disease primarily recruited by referral from US and Canadian rheumatologists. Being disease‐specific, its widespread use requires the assumption that the statistical mapping model is appropriate across disease areas. The second study, co‐ordinated by the EuroQol Group, was carried out in six countries and included eight patient populations,^2^ with a student cohort representing the healthy population. Although the dataset includes more than one disease area, it was not chosen to be representative of any specific population and thus suffers from the same problem of external validity as the FORWARD dataset. There are additional issues in terms of study design detailed in (Hernández‐Alava et al., 2017). Given these concerns about the currently available MI datasets and the importance of mapping for policy purposes, a large‐scale data collection designed for the purpose of mapping that can be used for decision making in the UK has been recommended (Hernández‐Alava et al., 2017).

Analysis by Hernández Alava et al. (2020, chapter 5.1) has produced evidence suggesting a low degree of empirical consistency between the orderings of health states by the 3L and 5L instruments when both are implemented within an MI survey. There are three obvious possibilities for the source of this inconsistency: conceptual (respondents interpret 3L and 5L as asking about different health concepts); or random reporting error (respondents are clear about the concept but make idiosyncratic “errors” in reporting); or repetition bias (response behavior is changed systematically by the experience of being asked similar questions earlier in the same interview).

The last of these could arise in several ways that are extensively discussed in the survey methods literature (see Schuman & Presser, 1996 for discussion of the effects of question ordering and context on response behavior). However, that literature is mostly concerned with the design of questionnaire modules containing a set of questions about specific aspects followed or preceded by an overall summary question (see McFarland, 1981, e.g.). Instead, we are concerned here with an issue that has had very little research attention: the ordering of two versions of a multi‐aspect instrument.

If MI survey responses are systematically distorted by behavioral effects induced by near‐repetition, then the use of statistical mapping based on such reference samples is open to question, and there is an urgent need to investigate this using appropriately designed experiments. More constructively, good experimental evidence on the behavior of respondents in MI surveys may give us a basis for recommending survey designs that improve the reliability of MI data.

In this study, we investigate the reliability of the MI survey design by developing and analyzing data from a randomized experiment designed to investigate whether inclusion of both versions of EQ‐5D at different stages in a single interview gives a reliable picture of the relationship between health measures based on the two instruments. We used the *Understanding Society* general population panel survey—also known as the UK Household Longitudinal Survey (UKHLS)—as the vehicle for our experiment.

## 2 METHODS

### 2.1 The experiment

Our experiment was carried in wave 11 of the UKHLS Innovation Panel, with fieldwork conducted in June to September 2018.^3^ The UKHLS is a nationally representative panel study with a two‐stage stratified random sample of the household population. It aims to interview every adult (aged 16 and over) household member annually, and the Innovation Panel is a subset of the full panel (2898 achieved adult interviews in 1856 households at wave 11) which is reserved for experimentation of various kinds (Jäckle et al., 2018).

Our experimental design randomized the sample of households into three approximately equal‐sized groups, and every individual within a household was allocated the same experimental treatment. The EQ‐5D instruments^4^ were embedded in the general individual interview (lasting approximately 20 min on average), which covered a wide range of socioeconomic topics. One group received the 5L module early (at approximately the halfway point in the interview) and the 3L module later (approximately three‐quarters of the way through the interview)^5^; the second group received the 3L instrument early and the 5L instrument later; and the third group received only the 5L instrument, at the later point in the interview. We refer to these groups henceforth as *5L3L*, *3L5L* and *5Lonly*. The two variants of EQ‐5D were separated by numerous survey questions, with a wide range of topics and response options. Not all respondents answered exactly the same questions, as some are context specific (e.g., retired individuals were not asked about job details).

Fieldwork used a multimode strategy, with a random two‐thirds of the panel initially offered a web interview (CAWI) and the remainder by computer‐assisted self‐interviewing (CASI) embedded within the main face‐to‐face interview.^6^ Assignment to interview mode groups was independent of assignment to EQ‐5D treatment groups, but individuals could opt for other interview modes and overall tended to prefer conventional interviews: 55% of achieved interviews were by CASI and 45% by CAWI.^7^

The achieved sample of individuals who gave full responses to the 5L (3L) instrument comprised 2635 (1732) individuals, clustered in 1689 (1278) households. We tested the balance of the sample across treatment groups using Fisher's exact test of independence, based on cross‐tabulations, constructed using only the first respondent individual from each household to avoid intra‐household correlation. We found no flaws in the randomization with respect to gender (*p* = 0.52), age group (*p* = 0.15) or existence of a limiting illness or disability (*p* = 0.37). The incidence of CAWI rather than CASI interviewing was also balanced across treatment groups (*p* = 0.95).

### 2.2 Sample characteristics

Since the UKHLS is a general population survey, many respondents reported no current health problems: the sample proportions of those reporting the best health state (11111) are 50% and 41% for 3L and 5L, respectively. However, the sample does have coverage of poorer health states. In their responses to a separate question, 36% of the sample reported a long‐standing illness or disability (LSI); among that group, the sample proportions of state (11111) fall to 17% (3L) and 14%. (5L) Appendix Figures A1 and A2 show the distributions of overall summary measures of 3L and 5L responses in the whole sample and the subset of respondents reporting a LSI. For the “misery” measure (the unweighted sum of the scores in each domain) the LSI group have 21% or 31% more misery than the sample average for the 3L and 5L instruments, respectively. Using the Dolan (1997) and Devlin et al. (2018) utility scores, the LSI group have 20% or 15% less utility on average.

The number of distinct health states reported by the 1732 respondents who completed 3L fully was 64 (26% of the 243 logically possible 3L states). For the 2635 respondents completing 5L, the number of distinct states reported was 269 (9% of the 3125 possible). These numbers compare with the 123 (51%) and 676 (22%) distinct states found for 3L and 5L in the EuroQol MI dataset and 136 (56%) and 960 (31%) for the Jan 2011 FORWARD dataset analyzed by Hernández and Pudney (2017). The number of distinct own health states reported by experimental subjects in the 5L valuation study of Devlin et al. (2018) was 180 (6%; Hernández‐Alava, Wailoo, et al., 2018). Thus the coverage of health states by UKHLS is less wide than that of disease‐linked MI datasets, but it is comparable to or more extensive than coverage by other datasets used for valuation purposes.

### 2.3 Analytical methods

We analyze the data in five steps. First, we examine the awareness by respondents of the repetition. As part of our design, CASI interviewers were asked to assess the reactions of respondents in the *5L3L* and *3L5L* groups to receiving repeated EQ‐5D instruments, distinguishing between those who appeared not to notice the repetition, those who noticed but appeared unconcerned, and those who expressed dissatisfaction or other concern about the repetition. We use ordinal response models to examine whether awareness is uniform across respondent groups.

Second, we test the hypothesis of equal response distributions across the *3L5L*, *5L3L* and *5Lonly* groups, using domain‐specific χ^2^ tests and a joint test across all domains, applied to each pair of treatment groups. The joint test is useful as it avoids the pitfalls of multiple testing, but the domain‐specific tests are valuable to identify the sources of significant overall difference. Define **p**_g_ as the vector of sample proportions from group *g* ∈ {*3L5L*, *5L3L*, *5Lonly*} of the first *k*‐1 response options, where *k* is the number of levels (3 or 5). The test statistic for comparison of groups *g* and *h* is: $\chi^{2}={\left(p_{g}–p_{h}\right)}^{\prime }{\left[(\text{diag}\left(p_{g}\right)-p_{g}{p_{g}}^{\prime })/n_{g}+(\text{diag}\left(p_{h}\right)-p_{h}{p_{h}}^{\prime })/n_{h}\right]}^{-1}\left(p_{g}–p_{h}\right)$. Under the assumption of strictly positive population probabilities and the null hypothesis of identical response probabilities, this has a limiting *χ* ^2^(*k*‐1) distribution as n_g_, *n*_*h*_ → ∞ with *n*_*g*_/*n*_*h*_ → constant. For some domains there were no responses at the bottom of the response scale. In these cases, we apply the test with the bottom two categories merged and the degrees of freedom therefore reduced by 1. Asymptotically, the test remains valid when implemented in this way, whether or not the population probability of the bottom category is strictly positive. We use only one respondent per household (the first one interviewed), to ensure independence.

The third step is to examine the prevalence of low‐3L/high‐5L or low‐5L/high‐3L conflicts within the *3L5L* and *5L3L* groups, using binary probit models to identify types of respondent particularly prone to such conflicts.

Although instruments like EQ‐5D are designed for practical policy use, research on health outcome measurement often stops short of examining the impact of measurement differences in practice. In fact, statistically significant differences between the three experimental groups may or may not matter for practical purposes, so our fourth step is to examine the impact of between‐group differences on statistical mapping, which is the main practical use of MI surveys. We estimate separate statistical mapping models using data from the *3L5L* and *5L3L* experimental groups and test the hypothesis that the resulting mapping functions are identical. The Dolan (1997) utility tariff allocates utility scores (*U*_3_) in the range −0.594 to 1, where 1 indicates the best possible level of health‐related quality of life and negative values indicate a state perceived as worse than dead. The distribution of 3L utility scores for the *5L3L* and *3L5L* groups combined and for the subsample of people with a LSI are shown in Figure A1a, 1b of the Appendix. The distribution is highly skewed, there is a discrete probability mass at the extreme value *U*_3_ = 1, and there are large gaps in the distribution—most importantly the absence of any utility scores in the interval (0.883, 1). A flexible approach to modeling is required if the distribution of 3L utility scores is to be captured adequately and models based on mixtures of generalized Tobit‐type components have been found to work well.^8^ We use the implementation of this idea developed by Hernández and Wailoo (2015), which involves a set of components labeled *c* = 1 … *C*:

(1) $$y_{ic}^{*}=\left\{\;\begin{array}{cc} 1 & \text{if}\;x_{i}\beta_{c}+\varepsilon_{ic}>0.883\\ x_{i}\beta +\varepsilon_{i}\; & \text{if}\;0.883\geq x_{i}\beta_{c}+\varepsilon_{ic}>-0.594\\ -0.594 & \text{if}\;x_{i}\beta_{c}+\varepsilon_{ic}\geq -0.594 \end{array}\right.$$

where $x_{i}$ is a vector of predictor covariates, $\beta_{c}$ is a coefficient vector and $\varepsilon_{ic}∼N\left(0,\sigma_{c}^{2}\right)$ a Gaussian random error, specific to component *c*. The probability of individual *i* belonging to the *c‐*th latent sub‐population has multinomial logit form:

(2) $$\mathrm{Pr}\left(\text{member}\;\text{of}\;c|w_{i}\right)=\frac{\exp\left(w_{i}\delta_{c}\right)}{\overset{C}{\underset{j=1}{\sum }}\exp\left(w_{i}\delta_{j}\right)}$$

where $w_{i}$ is a vector of covariates and one of the coefficient vectors $\delta_{1}\text{…}\;\delta_{C}$ is normalized to zero. The utility score $U_{i}$ for the observed 3L outcome is then assumed to be:

(3) $$U_{i}=\frac{y_{ic}^{*}\;\text{with}\;\text{probability}\;\exp\left(w_{i}\delta_{c}\right)}{\overset{C}{\underset{j=1}{\sum }}\exp\left(w_{i}\delta_{j}\right)}$$

Estimation of this structure is by maximum likelihood, and a combination of the Bayesian Information Criterion, mean absolute error, root mean squared error and graphical methods (Wailoo et al., 2017) are used to choose the number of components. Using a flexible model ensures that any differences in the mapping models are due to differences in the data (survey design) rather than model misspecification.

The fifth strand of our analysis summarizes the practical importance of differences between the mapping functions estimated from the *3L5L* and *5L3L* groups by illustrating the different results they produce in simple cost‐effectiveness calculations based on data from two real‐world clinical trials. The intention here is not to revise or make comparisons with the results of those studies, but to summarize the differences between our *3L5L* and *5L3L* experimental groups using a metric that is directly relevant to policy applications.

## 3 RESULTS

### 3.1 Do interviewees notice repetition?

Among the 863 respondents who received both 3L and 5L by CASI and for whom there is complete personal data, the reactions to receiving repeated EQ‐5D instruments were found to be independent of gender and the 3L/5L ordering.^9^ But, as Table 1 shows, there was a large age difference in the reactions, with people aged over 60 more than twice as likely as those aged under 35 to express awareness and concern over the repetition.

**TABLE 1:** Reactions to repetition of EQ‐5D module within age groups

Interviewer‐assessed reaction to repetition	Age group		
	16–34	35–59	60 and over
Did not appear to notice repetition	76.8%	57.2%	49.5%
Noticed repetition	11.6%	19.4%	23.8%
Noticed repetition and expressed concern	11.6%	23.4%	26.7%
Sample number in age group	181	367	315

Table 2 gives estimates, based on separate ordered probit models for the *5L3L* and *3L5L* groups, of the estimated impacts on the probabilities of reactions to repetition of a wider range of personal characteristics. The large significant age effects are confirmed, but reactions to repetition are unrelated to gender or the individual's health status, as represented by self‐reported LSI. There is no significant evidence of a difference between the *5L3L* and *3L5L* groups in respondents' awareness of the repetition. This might be seen as surprising, since the *5L3L* group involves following a detailed instrument (5L) by a less detailed one (3L) which could seem less justifiable to respondents than the reverse ordering used in the *3L5L* group.

**TABLE 2:** Estimated marginal effects on probabilities of reactions to repetition of EQ‐5D module (ordered probit model)

Covariate	*5L3L* group		*3L5L* group	
	*Pr* (noticed, unconcerned)	*Pr* (noticed with concern)	*Pr* (noticed, unconcerned)	*Pr* (noticed with concern)
LSI	−0.001 (0.011)	−0.002 (0.033)	−0.001 (0.011)	−0.003 (0.036)
Age 35–59	0.065*** (0.024)	0.121*** (0.036)	0.058*** (0.021)	0.125*** (0.037)
Age over 60	0.081*** (0.024)	0.179*** (0.039)	0.069*** (0.022)	0.174*** (0.044)
Female	0.010 (0.011)	0.029 (0.032)	−0.002 (0.010)	−0.008 (0.033)

*Note*: Standard errors adjusted for clustering by household.

Coefficient significance: * = 10%, ** = 5%, *** = 1%.

### 3.2 Are EQ‐5D responses significantly affected by timing and sequencing?

Table 3 reports the results of domain‐specific *χ* ^2^ tests of the hypothesis of equal response distributions between pairs of treatment groups, and the overall *χ* ^2^ test spanning all five EQ‐5D domains. We apply these tests separately to the subsamples of people who do not report a LSI and those who do (and who may therefore be more representative of participants in some clinical trials).

**TABLE 3:** *χ* ^2^ tests for equality of response distributions across treatment groups by domain of EQ‐5D (*p*‐values in parentheses)

Comparison	Sample group^a^	Domain						Joint test all domains^c^
		Mobility	Self‐care	Activities	Pain	Anxiety		
Equality of 3L response distributions: χ^2^(2) statistic								
*5L3L* versus *3L5L*	No LSI	0.14	2.94*	1.37	2.04		4.53	9.24
		[0.708]^b^	[0.086]^b^	[0.242]^b^	[0.153]^b^		[0.104]	[0.161]
	LSI	5.45**	3.98	2.24	8.08**		0.32	17.20**
		[0.020]^b^	[0.137]	[0.326]	[0.018]		[0.852]	[0.046]
Equality of 5L response distributions: χ^2^(4) statistic								
*5L3L* versus *3L5L*	No LSI	4.04	2.08	2.00	31.89***		9.38	49.02***
		[0.257]^b^	[0.555]^b^	[0.572]^b^	[0.000]^b^		[0.522]	[0.000]
	LSI	6.83*	6.63*	2.03	19.54***		2.62	33.42**
		[0.078]^b^	[0.085]^b^	[0.729]	[0.001]		[0.624]	[0.015]
*5L3L* versus *5Lonly*	No LSI	2.67	4.10	2.07	2.69		1.42	14.75
		[0.445]^b^	[0.251]^b^	[0.559]^b^	[0.442]^b^		[0.840]	[0.543]
	LSI	5.01	1.88	3.77	10.54**		2.23	20.49
		[0.171]^b^	[0.598]^b^	[0.439]	[0.032]		[0.694]	[0.306]
*3L5L* versus *5Lonly*	No LSI	4.92	1.60	0.96	18.98***		14.04***	33.80***
		[0.178]^b^	[0.660]^b^	[0.810]^b^	[0.000]^b^		[0.007]	[0.006]
	LSI	3.49	6.67*	1.86	2.10		0.85	15.79
		[0.479]	[0.083]^b^	[0.761]	[0.717]		[0.931]	[0.671]

^a^ Sample of first‐interviewed member in each household. LSI = group members reporting a long‐standing illness or disability.

^b^ Indicates degrees of freedom reduced from 2 to 1 for 3L domains or from 4 to 3 for 5L domains (see footnote 5).

^c^ Degrees of freedom for the joint test is the sum of the degrees of freedom in the five domain‐specific tests.

Statistical significance: * = 10%, ** = 5%, *** = 1%.

The overall *χ* ^2^ test for responses to the simpler 3L instrument finds no significant evidence of a difference between the *5L3L* and *3L5L* groups for respondents without any LSI. However, among people who do report an LSI, we find a significant difference (at the 5% level) between the 3L response distributions. Domain‐specific tests suggest that the source of the differences are the mobility and pain domains.

Comparisons of the 5L response distributions reveal significant differences for *5L3L* versus *3L5L* (LSI and no‐LSI) and for *3L5L* vs*. 5Lonly* (no LSI).^10^ In all of these cases, the pain dimension is the primary source of the difference, and there is also some domain‐specific evidence of a difference in the pain responses for *5L3L* versus *5Lonly* (LSI). Only in one other case (*3L5L* vs*. 5Lonly*, anxiety/depression domain for non‐LSI respondents) is there a significant difference at the 5% level.

The pain dimension of EQ‐5D should clearly be the main focus of concern for consistency between measures, and this tends to confirm the conclusions of consistency analysis of the existing FORWARD and EuroQol Group datasets (Hernández‐Alava & Pudney, 2017). The structure of the Dolan (1997) utility tariff used in many UK cost‐effectiveness studies assigns relatively high weight to the pain dimension,^11^ which means that pain may often be pivotal in determining the outcome of cost‐effectiveness studies. The significant response difference we find for the pain domain is therefore a potentially important policy issue.

**FIGURE 1:**
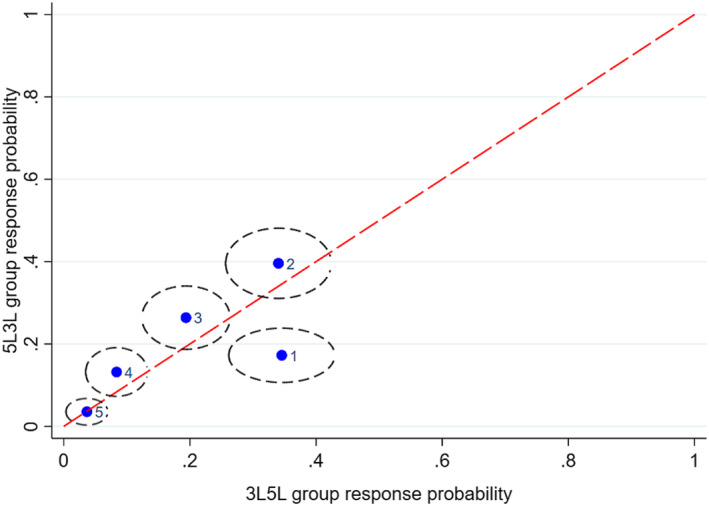
Sample proportions of responses to the 5L pain question in the *5L3L* and *3L5L* groups from respondents with long‐standing illness or disability (LSI; 95% confidence ellipses; labels: 1 = no pain, …, 5 = extreme pain)

**FIGURE 2:**
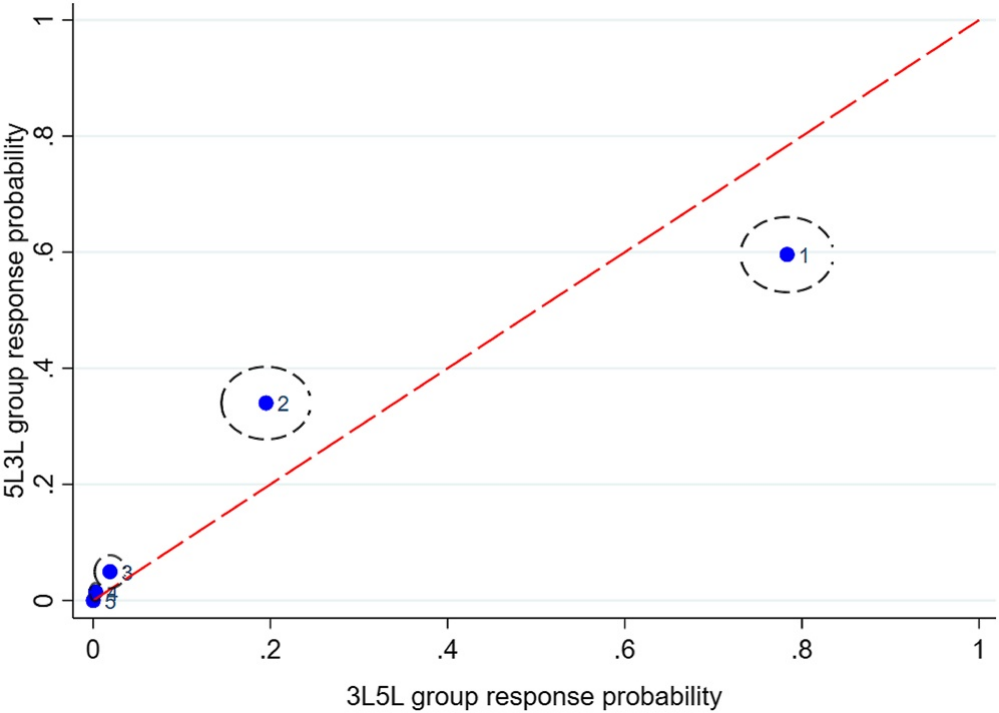
Sample proportions of responses to the 5L pain question in the *5L3L* and *3L5L* groups from respondents with no long‐standing illness or disability (LSI; 95% confidence ellipses; labels: 1 = no pain, …, 5 = extreme pain)

**FIGURE 3:**
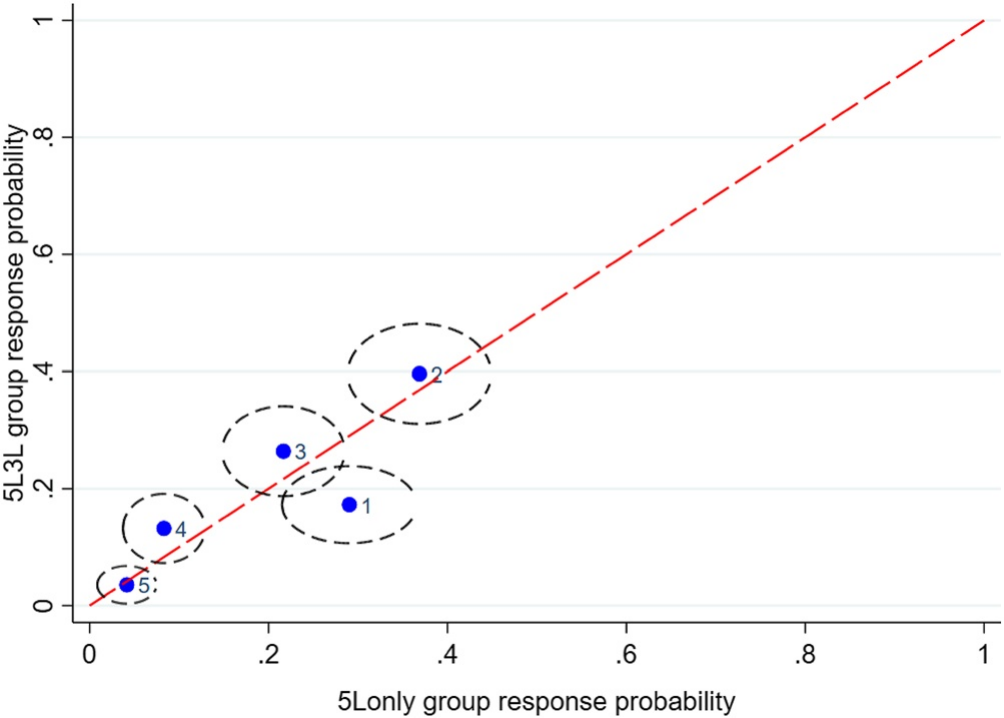
Sample proportions of responses to the 5L pain question from respondents with long‐standing illness or disability (LSI) in the *5L3L* and *5Lonly* groups (95% confidence ellipses; labels: 1 = no pain, …, 5 = extreme pain)

**FIGURE 4:**
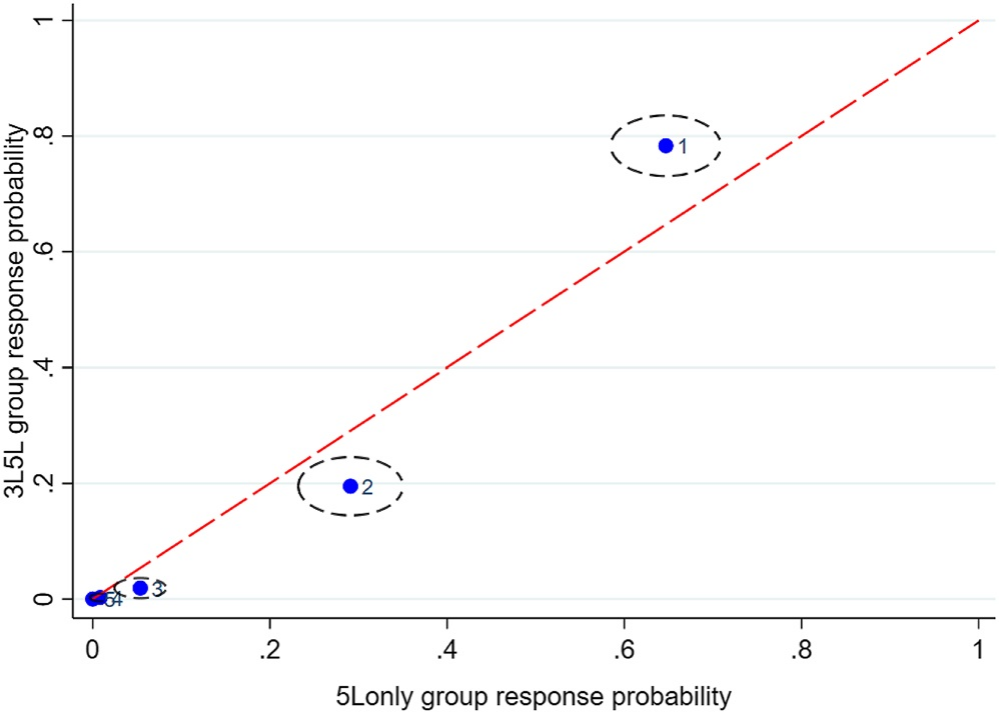
Sample proportions of responses to the 5L pain question from respondents with no long‐standing illness or disability (LSI) in the *3L5L* and *5Lonly* groups (95% confidence ellipses; labels: 1 = no pain, …, 5 = extreme pain)

**FIGURE 5:**
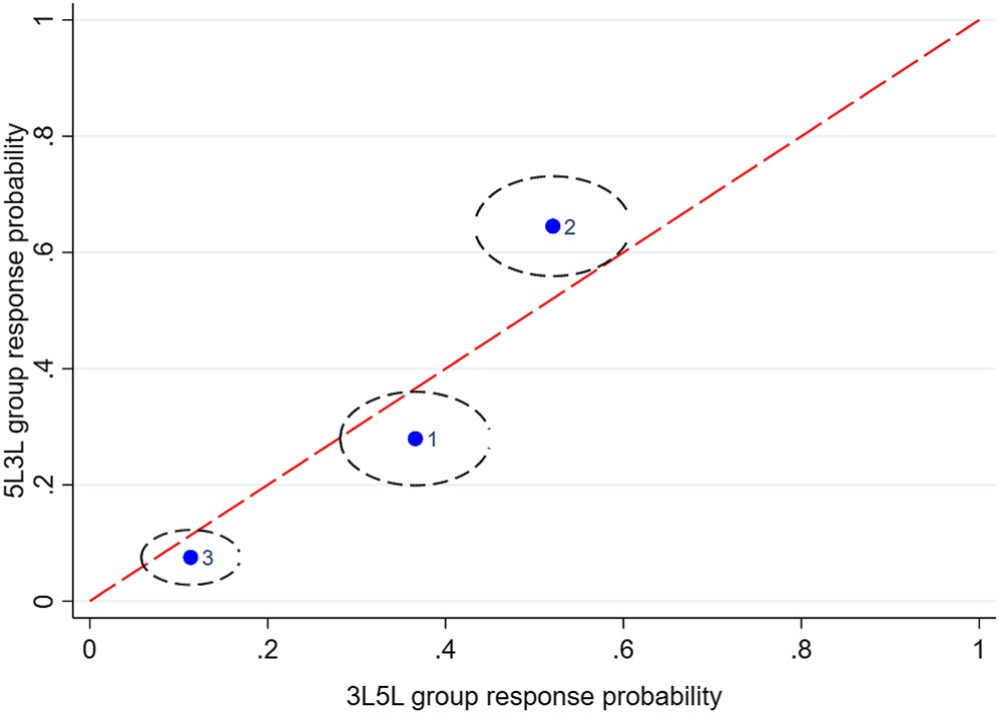
Sample proportions of responses to the pain dimension of 3L by respondents with long‐standing illness or disability (LSI) in the *5L3L* and *3L5L* groups (95% confidence ellipses; labels: 1 = no pain, …, 3 = extreme pain)

Figures 1, 2, 3, 4, 5 show the direction of the differences between the group‐specific response proportions in the pain domain, for those cases where the *χ* ^2^ test led to a result significant at the 5% level.^12^ We begin with responses to the 5L instrument.

Figures 1 and 2 compare the *5L3L* and *3L5L* groups, for LSI and no‐LSI respondents, respectively. The *5L3L* group gives a significantly lower sample proportion for response 1 (no pain) than the *3L5L* group, and thus paints a more negative picture of pain. A possible behavioral interpretation of this difference relates to two factors, sequencing and timing. It is well‐established in the survey methods literature that response behavior can be influenced by the existence and nature of questions asked earlier, so the different sequencing of questions in the *5L3L* and *3L5L* groups may generate behavioral differences. For example, the *3L5L* group may deliver a relatively positive assessment because 5L is preceded by 3L and the shorter response scale of 3L has the effect of sensitizing respondents particularly to the upper part of the 5‐point scale for 5L.

The timing of a question may also be an important influence because respondents learn or become fatigued or disengaged as the interview progresses, so that questions generate different responses if asked late in the interview than they would if asked early. A possible interpretation alternative or complementary to one based on sequencing is that the later timing of 5L finds respondents in the *3L5L* group more fatigued or disengaged and thus more likely to check the first box they see in the response scale, which happens to be “no pain.” This explanation is certainly consistent with the evidence of Figures 1, 2, 3 but not, on its own, with Figures 4 and 5.

Figures 3 and 4 show the other significant differences in 5L pain responses. Figure 3 (*5L3L* and *5Lonly* respondents with LSI), shows a worse pain state on average for the former group, while Figure 4 (*3L5L* and *5Lonly* respondents with no LSI) shows a worse pain state in the latter group. Since *5L3L* and *5Lonly* differ only in terms of timing and *3L5L* and *5Lonly* differ only in terms of the existence of a preceding 3L instrument, the differences in Figures 3 and 4 are again consistent with the interpretation that later timing and previous exposure to the 3L instrument both tend to promote choice of the first “no pain” response point in 5L. Thus the significant between‐group differences in 5L responses are all consistent with a simple behavioral interpretation that runs in terms of sequencing and timing effects.^13^

Figure 5 compares the (significantly different) 3L responses from the *5L3L* and *3L5L* groups for people who reported having an LSI. As was the case for 5L responses, it reveals a lower proportion of “no pain” responses in the *5L3L* group than the *3L5L* group. However, this implies a different pattern in terms of sequence and timing than the pattern for 5L responses. Within the *3L5L* group, 3L is asked early and is not preceded by another variant of EQ‐5D. Consequently, the effect on 3L responses of preceding 3L by 5L is the mirror image of the effect on 5L responses of preceding 5L by 3L.

How should we interpret this puzzling finding? It may hinge on the look of the CASI or CAWI screen that shows the response scale to respondents (see Figure A7 in the appendix). We have found that 5L preceding 3L tends to promote use of lower points (worse pain states) in the 3L response scale, while 3L preceding 5L tends to promote use of the upper points (better pain states) in the 5L scale. The 3L screen has a shorter scale that occupies the upper part of the screen, and perhaps sensitizes the respondent to the upper part of the 5L response scale when it eventually appears. Conversely, the longer 5L scale stretches deeper down the screen and perhaps sensitizes the respondent to the lower part of the 3L scale when it appears. If this interpretation is correct, it is remarkable, since there is a substantial gap between the early and late placement of the instruments. It would also have important implications for the design of MI surveys, giving some grounds for use of randomized mixtures of ascending and descending or horizontal and vertical display of response scales.

Table 4 shows the nature of these response differences in terms of average differences between treatment groups in overall summary health measures constructed from the EQ‐5D responses. Although the sample is balanced across treatment groups, we estimate the treatment effects using the inverse probability weighting estimator, adjusting for differences with respect to age and gender. The two summary measures are a utility score (taken from Dolan, 1997 for 3L and Devlin et al., 2018 for 5L) and the “misery” index formed by summing numerical responses to the five EQ‐5D items and rescaling to put the sum on the [0,1] interval.

**TABLE 4:** Average treatment effects for utility score and misery index summary measures (inverse probability weighting estimators with age group and gender covariates)

Treatment group	3L		5L	
	Utility score	Misery index^a^	Utility score	Misery index^b^
Respondents with no long‐standing illness or disability				
*3L5L* versus *5L3L*	0.002 (0.008)	−0.005 (0.005)	0.018*** (0.005)	−0.016*** (0.004)
*5Lonly* versus *5L3L*			−0.005 (0.006)	0.004 (0.005)
Respondents reporting a long‐standing illness or disability				
*3L5L* versus *5L3L*	0.024 (0.024)	−0.040*** (0.015)	0.059*** (0.020)	−0.046*** (0.016)
*5Lonly* versus *5L3L*			0.041** (0.020)	−0.031** (0.016)

*Note*: All available observations, with standard errors adjusted for clustering by household.

^a^ Sum of 3L items minus 5, divided by 10.

^b^ Sum of 5L items minus 5, divided by 20.

Statistical significance: * = 10%, ** = 5%, *** = 1%.

### 3.3 Conflicts between 3L and 5L responses

Table 5 summarizes the sample proportions of cases in which there is evidence of conflict between the health states described by the 3L and 5L responses. For any one of the five health domains, we define a low‐3L, high‐5L conflict as any case where the 3L response is at 1 and the corresponding 5L report at 3 or more, or where the 3L response is 2 and 5L is 5.^14^ Such a conflict means that the 5L instrument generates an implausibly poor health state relative to 3L. A high‐3L, low‐5L conflict is a case where the 3L response is 3 and the 5L instrument gives 1‐3, or where the 3L response is 2 and 5L is 1.

**TABLE 5:** Sample proportions of conflicting 3L and 5L reports

Conflict	Domain	*5L3L* group	*3L5L* group
3L = 1 & 5L ≥ 3 *or* 3L = 2 & 5L = 5	Mobility	0.2%	0%
	Self‐care	0%	0%
	Activities	0.1%	0%
	Pain	0.4%	0%
	Anxiety	0%	0.2%
Any domain in low‐3L, high‐5L conflict^a^		**0.7**%	**0.2**%
		(0.2%–1.4%)	(0.1%–0.6%)
3L = 3 & 5L ≤ 3 *or* 3L = 2 & 5L = 1	Mobility	1.9%	1.4%
	Self‐care	0.9%	0.4%
	Activities	2.6%	0.8%
	Pain	0.8%	2.5%
	Anxiety	0.5%	1.2%
Any domain in high‐3L, low‐5L conflict^a^		**5.0**%	**5.2**%
		(3.5%–6.4%)	(3.8%–6.6%)

^a^ Bias‐corrected 95% bootstrap confidence intervals, clustered by households.

Table 5 shows that our experiment yielded very few cases where the 3L instrument indicated good health and the 5L instrument conflicting poor health. However, there were rather more cases (around 5% of the groups that received both 3L and 5L), where the 5L instrument gave an apparently conflicting good health state for at least one of the five domains. This approximately 5% conflict rate is identified with good precision by the experimental comparison and is similar for both treatment groups.

Although a 5% conflict rate may not appear very large, it could be large enough to cause substantial problems in certain types of statistical modeling^15^ and, when converted to utility form, in cost‐effectiveness calculations.

Table 6 shows the results of probit models for the occurrence across individual respondents of any type of 3L‐5L conflict. The first model is based on the whole sample from the *5L3L* and *3L5L* groups and allows for the impact of web interviewing on the conflict rate; the second model restricts the sample to respondents interviewed by CASI for whom interviewers could record whether repetition appeared to have been noticed by the respondent. We find a strong impact of health, with LSI raising the rate of conflicting responses by approximately 4 percentage points. There is statistically significant evidence of a lower rate of conflict (by 2.4 percentage points) in web interviews, which may reflect the characteristics of respondents willing to use the web rather than the CAWI mode itself. There is only weak evidence (*p* = 0.111) of a relationship between conflicting responses and awareness of repetition, although the point estimate is large—an average increase of 2.4 percentage points in the conflict rate for respondents who show no sign of awareness of the repetition. There are no significant differences between the *5L3L* and *3L5L* groups or between demographic groups.

**TABLE 6:** Marginal effects on the probability of conflicting 3L and 5L responses (probit models)

Covariate	Combined *5L3L* and *3L5L* sample	CASI subsample, combined *5L3L* and *3L5L*
LSI	0.047***	0.038***
	(0.013)	(0.015)
Age 35–59	−0.008	−0.010
	(0.014)	(0.016)
Age 60+	−0.002	0.002
	(0.015)	(0.018)
Female	0.003	0.002
	(0.011)	(0.013)
Web interview	−0.024**	
	(0.011)	
*3L5L* group	0.006	0.003
	(0.011)	(0.013)
Repetition unnoticed		0.024
		(0.015)

*Note*: Standard errors in parentheses, adjusted for clustering by households.

Statistical significance: * = 10%, ** = 5%, *** = 1%.

## 4 BUT DOES IT MATTER? GROUP DIFFERENCES EXPRESSED IN A COST‐EFFECTIVENESS METRIC

The comparison of responses between treatment groups suggests that there are significant biases in EQ‐5D measures produced by repetitive MI surveys. These distortions have the potential to induce measurement error bias into the predictive models that are used for mapping between 3L and 5L quality of life measures. But a statistically significant bias is not necessarily large enough to cause important problems for policy work. To give some insight into this, we use two recent cost‐effectiveness studies to illustrate the response differences revealed by the UKHLS experiment using a framework more directly related to practical applications.

### 4.1 Mapping models

The original 3L, combined with the utility scores constructed by Dolan (1997), currently remains the version preferred by the NICE as a basis for cost‐effectiveness work in England. For clinical trials which collect responses from trial subjects using the updated 5L instrument, mapping to the 3L instrument is currently mandated by NICE (2013). It is therefore of interest to use the impact on mapping models as a summary of the experimental group differences we have found.

A statistical mapping model predicts the 3L responses that would have been observed, had the 3L version been implemented, using the 5L responses as predictors. In our illustrative application, we use the Hernández‐Wailoo (2015) mixture model described in Section 2.3. After extensive specification search,^16^ the final vector of predictors $x_{i}$ contained an intercept and the five items of 5L representing mobility, self‐care, usual activities, pain and anxiety/depression, each treated as numerical variables on the scale 1 … 5. The predictor $w_{i}$ for group membership contained an intercept, a binary variable identifying individuals who reported the best possible health state 11111, and the 5L misery index formed from summing the domain indicators, giving a single variable with range 5 … 25.

Following a standard specification search carried out independently on the *5L3L* and *3L5L* samples, we arrived at a similar three‐component specification for each. Component *c* = 1 was a degenerate discrete probability mass at $U_{i}=1$, with two other regular Tobit‐type components. The parameter estimates are shown in Table 7, together with the predicted mean utility scores conditional on group membership and the mean membership probabilities.

**TABLE 7:** Parameter estimates of mixture models for 3L utility

Parameter	*5L3L* group			*3L5L* group		
	1	2	3	1	2	3
Component‐specific regression functions						
Constant	1	1.535***	0.978***	1	0.632***	0.991***
		(0.046)	(0.011)		(0.089)	(0.016)
Mobility	‐	−0.057*	−0.046***	‐	−0.030	−0.021***
		(0.023)	(0.010)		(0.022)	(0.006)
Self‐care	‐	−0.086***	−0.019*	‐	−0.043*	−0.011
		(0.024)	(0.009)		(0.022)	(0.014)
Usual activities	‐	−0.053*	−0.011	‐	0.012	−0.025***
		(0.026)	(0.013)		(0.014)	(0.007)
Pain	‐	−0.100***	−0.047***	‐	−0.090***	−0.058***
		(0.021)	(0.006)		(0.018)	(0.005)
Anxiety/depression	‐	−0.142***	−0.007	‐	−0.028	−0.018***
		(0.013)	(0.005)		(0.015)	(0.004)
$\sigma_{c}$	‐	0.174***	0.041***	‐	0.109***	0.052***
		(0.103)	(0.170)		(0.154)	(0.073)
Component membership multinomial logit coefficients						
Constant	8.045**	−2.265*	‐	−2.078	−6.537***	‐
	(3.123)	(1.155)		(3.190)	(0.603)	
Misery index	−1.504**	0.190*	‐	−0.151	0.492***	‐
	(0.553)	(0.0753)		(0.381)	(0.0605)	
Perfect health (state 11111)	17.74***	18.46***	‐	5.532***	1.484	‐
	(0.848)	(0.801)		(1.291)	(0.979)	
Mean predictions						
3L utility	1.000	0.847	0.799	1.000	0.366	0.806
Membership probability	0.366	0.290	0.344	0.406	0.087	0.507

*Note*: Standard errors in parentheses.

Statistical significance: * = 10%, ** = 5%, *** = 1%.

Although the models have the same three‐component structure, the estimates are quite different. A likelihood ratio test comparing the two separate models to the same structure fitted to the combined *5L3L* + *3L5L* sample gives a highly significant rejection of the hypothesis of equal parameters (*χ* ^2^(20) = 84.4, *p* = 1.4 × 10^−9^). The *5L3L* model has a probability mass of 0.37 at perfect health state *U* = 1 and two Tobit components with similar mean predictions (0.799 and 0.847) but with error variance differing by a factor of (0.174/0.041)^2^ = 18.0. The *3L5L* model has a slightly larger discrete probability mass at *U* = 1 and two other components with quite distinct mean predictions (0.806 and 0.366) and error variances that differ by a smaller margin of (0.109/0.052)^2^ = 4.4. In summary, it is clear that the significant differences we found in the *5L3L* and *3L5L* data on EQ‐5D have significant and structurally important implications for mapping models estimated from those data.

### 4.2 Cost‐effectiveness calculations

We put the differences in the mapping functions estimated from the *3L5L* and *5L3L* treatment groups into policy‐relevant form using two illustrative cost‐effectiveness applications. These cannot claim to be representative of the broad range of such studies considered by NICE, but they serve to illustrate the way that measurement differences may affect the key calculations made in cost effectiveness studies. The two applications meet our requirements in that they are based on individual‐level trial data incorporating the newer 5L version of EQ‐5D, and their authors kindly agreed to re‐run the cost‐effectiveness calculations for us. It is important to note that the results of these recalculations are simplified and not comparable with the authors' own findings—they relate to a small part of the evaluation process and are based on different mapping approaches. It should also be noted that they are both trials of interventions in health conditions that normally affect quality, rather than length, of life.

The TITRATE randomized control trial (RCT) was concerned with the management of established moderate cases of Rheumatoid Arthritis receiving stable doses with conventional drug therapies. The trial compared the outcomes of intensive case management (monthly assessments and support by trained nurses) in 169 cases with outcomes in a control group of 167 cases receiving standard care. The aim was to test the hypothesis that intensive management increases the rate of remission in such patients. The study was implemented in 39 English rheumatology centers. Health utilities were derived from participant responses to 5L at baseline, 6 and 12 months. Details of the trial and the economic evaluation are reported in Scott et al. (2020).

The HubBLe trial was a two‐arm RCT conducted in 17 acute UK hospitals from September 2012 to August 2015. The trial recruited a total of 372 patients with grade II or early grade III hemorrhoids (piles that prolapse but either spontaneously reduce or require minimal manual replacement). Patients were randomly assigned to either Haemorrhoidal Artery Ligation (HAL, *n* = 185) or more costly Rubber Band Ligation (RBL, *n* = 187) and followed for up to 12 months. The 5L measurements were made at baseline, 1 day, 7 days, 21 days, 6 weeks, and 12 months. Full details of the trial are reported by Brown et al. (2016) and an economic evaluation conducted alongside the trial is reported in Alshreef et al. (2017).

Let treatment 1 be the basic treatment (often the clinical status quo), and treatment 2 an alternative treatment hypothesized to be clinically superior. In outline, a cost‐effectiveness study involves the following steps:

(i) For each trial participant *j*, use 5L to measure the sequence of quality‐of‐life outcomes over time the trial period.
(ii) For each 5L measurement, use the mapping model to predict the expected value of the corresponding unobserved 3L utility outcome.
(iii) Convert the resulting sequence of predicted 3L quality‐of‐life measures to the metric of quality‐adjusted life years (QALY), $Q_{j}$.
(iv) Measure the total cost of treatment, $T_{j}$ for each participant as a present value over the trial period.
(v) Calculate the differences in mean cost and QALY outcomes between participants receiving treatments 1 and 2 as $\Delta T={\bar{T}}_{2}-{\bar{T}}_{1}$ and $\Delta Q={\bar{Q}}_{2}-{\bar{Q}}_{1}$.
(vi) Calculate the Incremental Cost‐Effectiveness Ratio (ICER), giving the cost per additional QALY delivered by treatment 2 beyond the benefit achieved with treatment 1:

$$\text{ICER}=\;\frac{\Delta T}{\Delta Q}$$

Treatment 2 is then recommended as an alternative to treatment 1 if the ICER is below the level that NICE regards as an affordable cost (normally in the region of £20,000–30,000 per additional QALY). If the ICER is above this threshold, then it is regarded as not cost‐effective, even if it offers a significantly better outcome in QALY terms.

Table 8 summarizes the results of re‐doing the cost‐effectiveness calculations for TITRATE and HubBLe,^17^ using the two alternative mapping models estimated separately from UKHLS *5L3L* and *3L5L* experimental data.

**TABLE 8:** HubBLe and TITRATE cost‐effectiveness studies

	Mapping based on *5L3L* data	Mapping based on *3L5L* data		Ratio *5L3L*/*3L5L*
*TITRATE* cost‐effectiveness study				
Usual care: mean cost	£2258		£2258	
	(156)		(156)	
Usual care: mean QALY	0.6312		0.5582	1.131
	(0.0163)		(0.0170)	
Intensive management: mean cost	£3784		£3784	
	(222)		(222)	
Intensive‐usual mean QALY	0.6590		0.5840	1.128
	(0.0145)		(0.0161)	
Intensive‐usual mean cost difference	£1526		£1526	
	(271)		(271)	
Intensive‐usual mean QALY difference	0.0279		0.0258	1.080
	(0.0218)		(0.0223)	
ICER for intensive versus usual management	£54,734		£59,124	0.926
*HubBLe* cost‐effectiveness study				
RBL mean cost	£709^a^		£709^a^	
	(95)		(95)	
RBL mean QALY	0.877		0.844	1.039
	(0.015)		(0.017)	
HAL mean cost	£1767^a^		£1767^a^	
	(101)		(101)	
HAL mean QALY	0.885		0.850	1.041
	(0.015)		(0.017)	
HAL‐RBL mean cost difference	£1073		£1073	
	(190)		(190)	
HAL‐RBL mean QALY difference	0.0147		0.0140	1.050
	(0.0211)		(0.0246)	
ICER for HAL versus RBL	£73,009		£76,858	0.950

*Note*: Standard errors in parentheses relate to sampling variation in the trial data only; they do not take account of statistical error in the parameters of the model used to predict 3L values.

^a^ Mean costs are reported as descriptive statistics based on the complete case set of costs with *n* = 103 and *n* = 99 for RBL and HAL, respectively (Alshreef et al., 2017).

In the TITRATE trial, use of the *5L3L* mapping rather than the *3L5L* mapping gives a 13% higher estimated mean QALY for both the standard and intensive treatments. However, because the impact is fairly uniform across the two treatments, the QALY difference between the two treatments is only 8% higher for the *5L3L* mapping than the *3L5L* mapping. This in turn means that the estimated ICER is lower by about 7% when the *5L3L* mapping is used.

In the HubBLe trial, the *5L3L* mapping again leads to higher QALY outcomes than the *3L5L* mapping, although only by 4% for both the RBL and HAL treatments. The HAL‐RBL QALY difference is 5% higher for the *5L3L* mapping, giving an estimated ICER lower by 5%: approximately £77,000 rather than £73,000, but still well above the NICE funding threshold.

Both TITRATE and HubBLe share a feature that is likely to be found in a wide range of applications. Although error in the mapping procedure may generate large biases in the mean QALY estimates ${\bar{Q}}_{1}$ and ${\bar{Q}}_{2}$, those biases tend to cancel out to some degree, giving a smaller range of error in the QALY difference ΔQ. However, against this, the QALY difference ΔQ is the denominator of the ICER and, in evaluations where a new technology offers only marginal improvements, that denominator may be quite small. In such cases, even small biases in ΔQ may have a large impact on ICERs and thus on cost‐effectiveness decisions.

The HubBLe example also illustrates a further important point—that the type of mapping model used and the dataset it is based on may have a much bigger impact on the ICER than the response errors induced by repetitive interviewing in a MI survey. The published cost‐effectiveness result for HubBLe uses a simpler mapping model devised by van Hout et al. (2012) than our Tobit‐mixture model. That mapping led to an estimated ICER of £ 90,688 in the complete case analysis (Alshreef et al., 2017). That disagreement is much larger than the difference between the ICERs for the *5L3L* and *3L5L* group‐specific mappings (£73,009 and £76,858).

## 5 CONCLUSIONS AND IMPLICATIONS FOR FUTURE RESEARCH

There are many survey instruments used to measure health‐related quality of life outcomes as a basis for the evaluation of medical procedures and pharmaceutical products. Statistical mapping often has to be used to translate outcomes measured by one instrument into an evaluation metric developed in relation to a different instrument. This process requires a multi‐instrument survey that allows the statistician to observe responses to both instruments from the same set of survey participants. It also entails a reliability assumption: that participants give essentially the same responses in a multi‐instrument interview as they would in a conventional interview involving only one of the instruments. Despite the importance of statistical mapping for the policy analysis carried out by official bodies like NICE in England, there has so far been little research aiming to test the hypothesis that multi‐instrument surveys are reliable in this sense.

In this paper, we have exploited a randomized experiment in the UKHLS Innovation Panel incorporating two variants of the widely used EQ‐5D instrument (3L and 5L) differing primarily in the number of levels in the response scales. The experiment compared three treatment groups: 5L followed later in the interview by 3L; 3L followed by 5L; and 5L alone; to investigate the MI reliability hypothesis and explore its consequences for economic evaluation. There are seven main findings.

First, despite a relatively large separation in time between the two instruments in the interview, there was evidence that a majority of respondents were aware of the (near‐)repetition, with older people more likely to notice and express concern. This may be because older people were more engaged with the survey, rather than any inherent age difference in attention span or recall.

Second, there were statistically significant differences in both the 3L and 5L response distributions between *5L3L* and *3L5L*, so the ordering of the two instruments has a significant impact. There were also significant differences between the comparisons of the *5L3L* and *3L5L* groups with the *5Lonly* group. The strongest evidence of difference was found in the EQ‐5D domain relating to pain, which is the most highly weighted domain in the most widely used utility scoring systems for the UK.

Third, for the 5L instrument, these significant differences at the level of individual components of EQ‐5D also carry over to overall summary measures of health‐related quality of life, specifically the “misery index” defined as the sum over the five health domains of EQ‐5D, and the utility score proposed by Devlin et al. (2018). Evidence of an impact of question ordering on summary measures constructed from the 3L responses is less clear‐cut.

Fourth, the nature of the ordering effects for questions on pain is quite complex. We have found that, if 5L is preceded by 3L, then it generates a higher proportion of favorable “no pain” responses relative to asking 5L alone or before 3L. Conversely, when 3L is preceded by 5L, use by respondents of the “no pain” option is reduced relative to the responses from people who are asked 3L first. A possible interpretation is that asking 3L (with its short response scale) first visually primes respondents to the first few points on a subsequently encountered 5L scale; while asking 5L (with its longer scale) primes respondents to use later points on a 3L scale encountered subsequently.

Fifth, strongly conflicting responses (mostly where 3L indicated very poor health and 5L indicated very good health) occurred for just over 5% of respondents—a conflict rate that could be large enough to cause serious bias in some types of statistical analysis. The occurrence of conflicting responses is significantly higher among those with a long‐standing health problem, and is significantly lower among people completing the interview online.

Sixth, we used the 3L and 5L responses to estimate flexible statistical models for mapping from 5L measurements to the 3L utility basis approved by NICE for its cost‐effectiveness work. This was done separately for randomized groups with 5L preceding 3L and 3L preceding 5L, and we found that the fitted models exhibited substantial structural differences, suggesting that question ordering in MI surveys can have major implications for mapping procedures.

Seventh, we used the alternative *5L3L* and 3L5L mapping models to revisit two real‐life cost‐effectiveness studies, finding that the key ICER differed by 5% in one case and 7% in the other. Although there was no difference in the conclusions in either case (both ICER estimates were well above the £30,000 threshold often cited as the upper limit used by NICE), differences of this magnitude could be important for more marginal cost‐effectiveness applications. However, comparison with results obtained by the original study authors suggests that question ordering in the MI survey used to estimate the mapping model has much less effect than the choice of econometric approach used to develop the mapping model.

There are some important implications from our findings for the design of future multi‐instrument surveys to be used for mapping between health outcome measures. Most important is to be aware of the possibility that sequencing and timing of the 3L and 5L instruments within an interview may affect responses, and this means that there is a strong case for designs that randomize the ordering of the two instruments and possibly also randomize the use of ascending and descending response scales. Such designs would not “solve” the response error problem, but they would allow the analyst to check for the existence of response distortions and carry out robustness checks.

Many MI surveys used in health economics are now web‐based because of the lower cost of web interviewing. There is some concern that web surveys introduce bias through differences in the pattern of non‐response or changes in respondents' behavior. It is reassuring that we have found no evidence of significant differences between the CASI and CAWI subsamples, except in the probability of strongly conflicting 3L and 5L responses—where web interviews appear to reduce the frequency of conflict (and possibly thereby improve response quality).^18^

## CONFLICT OF INTEREST

No.

## Supporting information

Supplementary Material S1

## Data Availability

Data is available from University of Essex. Institute for Social and Economic Research. (2018). Understanding Society: Innovation Panel, Waves 1–10, 2008–2017. [data collection]. 9th Edition. UK Data Service. SN: 6849. http://doi.org/10.5255/UKDA‐SN‐6849‐10.

## ENDNOTES

^1^
The most comprehensive MI survey is that developed by the International Multi‐Instrument Comparison Project (https://www.aqol.com.au/index.php/aqol‐current), which carries no fewer than six multi‐attribute utility instruments, including 5L.

^2^
Cardiovascular disease, respiratory disease, depression, diabetes, liver disease, personality disorders, arthritis, and stroke

^3^
University of Essex. Institute for Social and Economic Research. (2018). Understanding Society: Innovation Panel, Waves 1–10, 2008–2017. [data collection]. 9th Edition. UK Data Service. SN: 6849 http://doi.org/10.5255/UKDA‐SN‐6849‐10

^4^
Tables A1 and A2 in the appendix reproduce the 5L and 3L questionnaires.

^5^
The FORWARD databank for rheumatoid arthritis and the EuroQol Group crosswalk survey both use 5L followed by 3L.

^6^
Appendix Table A3 reports the sample numbers by experimental treatment group and interview mode.

^7^
In an attempt to reduce non‐response, a late third round of telephone interviewing (CATI) was used to gain for non‐respondent household members where possible. This added only one additional interview (0.03% of the achieved sample).

^8^
An alternative approach, which we do not pursue here, is to estimate a multi‐dimensional ordinal model for mapping between the ten 3L and 5L questionnaire responses and then apply the utility tariff to predictions of the 3L items rather than predicting the 3L utility score directly; see Hernández and Pudney (2017).

^9^
In an ordered probit model for the three response categories, with gender, age groups and 3L/5L order used as covariates, gender and ordering were individually insignificant (*p* = 0.616 and 0.168, respectively) but the age variables were jointly significant (*χ* ^2^(2) = 29.8, *p* = 0.000).

^10^
Note that the *5L3L* versus *3L5L* and *3L* versus *5Lonly* comparisons are not statistically independent.

^11^
The effect of increasing pain from level 1 (“no pain”) to level 2 (“moderate”) is to reduce the utility score by 0.123; the next greatest impact is 0.104 for the self‐care domain. Increasing pain from level 1 to level 3 (“extreme”) reduces the utility score by 0.386; the next greatest impact is for mobility (0.314). The same is true for the utility tariff proposed by Devlin et al. (2018): increasing pain from level 1 to level 5 reduces utility by 0.335; the next greatest impact is 0.289 for anxiety/depression.

^12^
A full set of figures is included in the appendix Figures A3 to A6.

^13^
Note that the single other significant difference in the 5L responses (the anxiety/depression domain; *3L5L* vs. *5Lonly*; no LSI) is also consistent with this interpretation.

^14^
For the special case of the mobility domain, there was a change in the wording of the bottom response category, which may be responsible for some conflicts of type 3L = 2, 5L = 5: an individual may be unable to walk (5L = 5) but not be confined to bed (3L = 3).

^15^
See, for example, Hausman et al. (1998) for a Monte Carlo example where a 5% random misclassification of a binary dependent variable causes large biases in the estimation of a probit model.

^16^
We began the specification search with $x_{i}$ containing 20 binary indicators for each level of each domain and demographic variables, and $w_{i}$ containing the square as well as the level of 5L misery. Low cell counts for many of the 5L domain indicators prevented reliable estimation, and the alternative of treating the domain scales as continuous provided a good parsimonious fit to the data. Age and gender and misery squared were statistically insignificant and were dropped from the final model.

^17^
We use the complete case analysis reported as a sensitivity analysis in Alshreef et al. (2017).

^18^
However, it should be noted that the IP data used here comes from a long‐run panel of relatively engaged participants, most of whom will have been interviewed face‐to‐face in earlier waves. This conclusion may not carry over to all other web surveys.
