# Supplementary material for: Mapping between EQ‐5D‐3L and EQ‐5D‐5L: A survey experiment on the validity of multi‐instrument data
Source: Health Econ. 2022 Feb 28;31(6):923–39. doi: 10.1002/hec.4487 (PMC9303872; doi:10.1002/hec.4487)
Supplement: Supplementary file 1 — Supplementary Material S1 [file HEC-31-923-s001.docx]

**Mapping between EQ-5D-3L and EQ-5D-5L**

**A survey experiment on the validity of multi-instrument data**

**Appendix file**

**Appendix: Instrument wording and sample characteristics**

**Table A1** The 5L instrument

Under each heading, please tick the ONE box that best describes your health TODAY

*Mobility*

□ I have no problems in walking about

□ I have slight problems in walking about

□ I have moderate problems in walking about

□ I have severe problems in walking about

□ I am unable to walk about

*Self-Care*

□ I have no problems washing or dressing myself

□ I have slight problems washing or dressing myself

□ I have moderate problems washing or dressing myself

□ I have severe problems washing or dressing myself

□ I am unable to wash or dress myself

*Usual Activities (e.g. work, study, housework, family or leisure activities)*

□ I have no problems doing my usual activities

□ I have slight problems doing my usual activities

□ I have moderate problems doing my usual activities

□ I have severe problems doing my usual activities

□ I am unable to do my usual activities

*Pain / Discomfort*

□ I have no pain or discomfort

□ I have slight pain or discomfort

□ I have moderate pain or discomfort

□ I have severe pain or discomfort

□ I have extreme pain or discomfort

*Anxiety / Depression*

□ I am not anxious or depressed

□ I am slightly anxious or depressed

□ I am moderately anxious or depressed

□ I am severely anxious or depressed

□ I am extremely anxious or depressed

**Table A2**  The 3L instrument

By placing a tick in one box in each group below, please indicate which statements best describe your own health state today

*Mobility*

□ I have no problems in walking about

□ I have some problems in walking about

□ I am confined to bed

*Self-Care*

□ I have no problems with self-care

□ I have some problems washing or dressing myself

□ I am unable to wash or dress myself

*Usual Activities (e.g. work, study, housework, family or leisure activities)*

□ I have no problems with performing my usual activities

□ I have some problems with performing my usual activities

□ I am unable to perform my usual activities

*Pain / Discomfort*

□ I have no pain or discomfort

□ I have moderate pain or discomfort

□ I have extreme pain or discomfort

*Anxiety / Depression*

□ I am not anxious or depressed

□ I am moderately anxious or depressed

□ I am extremely anxious or depressed

| **Table A3** Sample numbers by experimental treatment group and interview mode | | | | | |
| --- | --- | --- | --- | --- | --- |
| Treatment group | CASI | CAWI | CATI | All modes | Complete EQ-5D responses |
| *5L3L* | 531 | 421 | 0 | 952 | 843 |
| *3L5L* | 518 | 443 | 0 | 961 | 862 |
| *5Lonly* | 547 | 435 | 1 | 983 | 887 |
| All groups | 1,596 | 1,299 | 1 | 2,896 | 2,592 |

| **Figure A1** Sample distributions of the 3L misery index and Dolan (1997) utility scores, whole sample and subset of respondents with long-standing illness or disability (LSI) | |
| --- | --- |
| 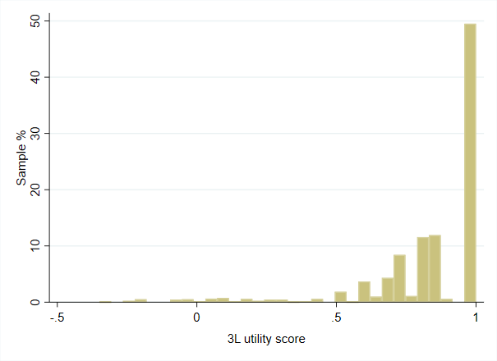 | 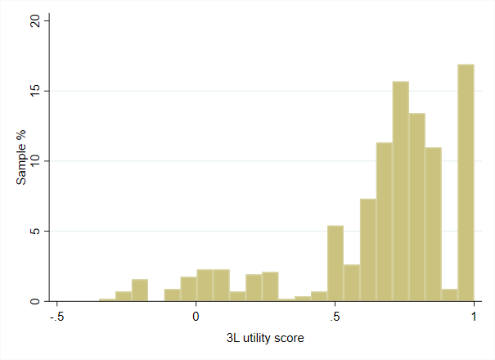 |
| 1. 3L utility score: whole sample | 1. 3L utility score: subset with LSI |
| 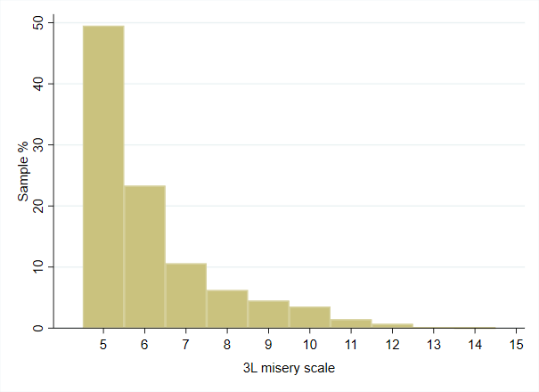 | 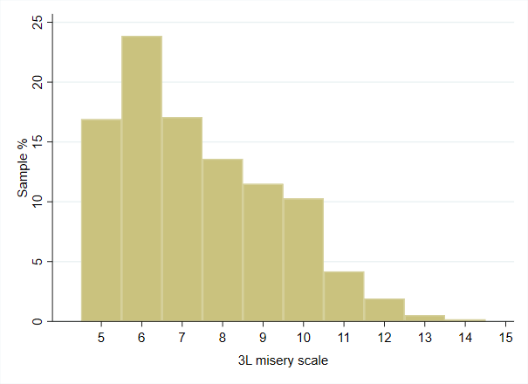 |
| 1. 3L misery index: whole sample | 1. 3L misery index: subset with LSI |

| **Figure A2** Sample distributions of the 5L misery index and Devlin *et al.* (2018) utility scores, whole sample and subset of respondents with long-standing illness or disability (LSI) | |
| --- | --- |
| 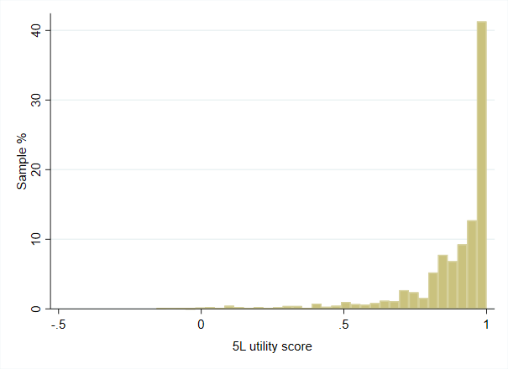 | 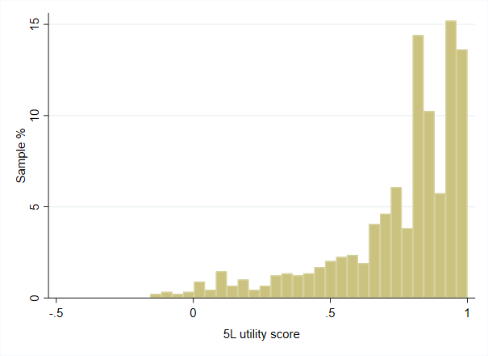 |
| 1. 5L utility score: whole sample | 1. 5L utility score: subset with LSI |
| 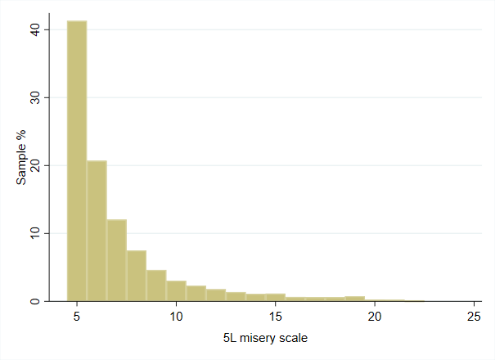 | 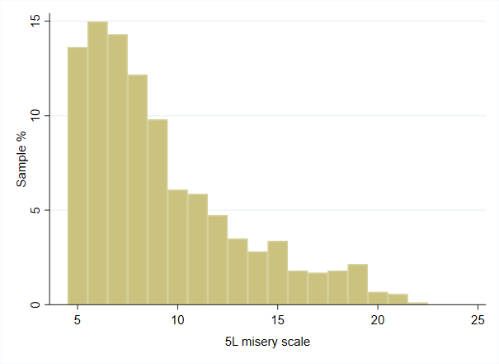 |
| 1. 5L misery index: whole sample | 1. 5L misery index: subset with LSI |

| **Figure A3** 5L response probabilities with 95% confidence ellipses: *5L3L* vs*.* *3L5L*, subsets of respondents without and with long-standing illness or disability (LSI) | |
| --- | --- |
| 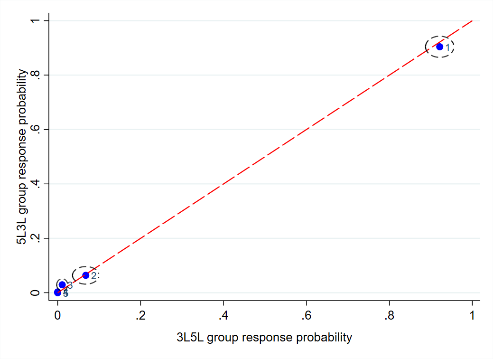 | 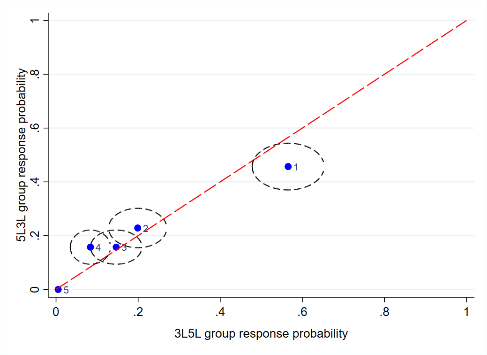 |
| 1. Mobility: subset with no LSI | 1. Mobility: subset with LSI |
| 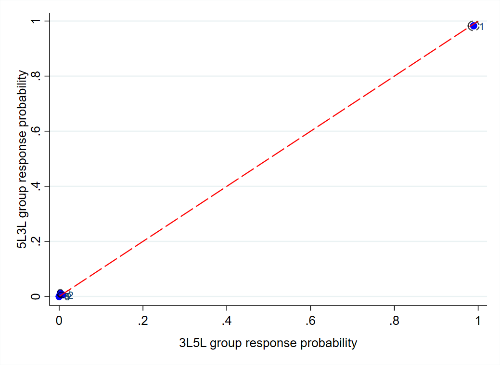 | 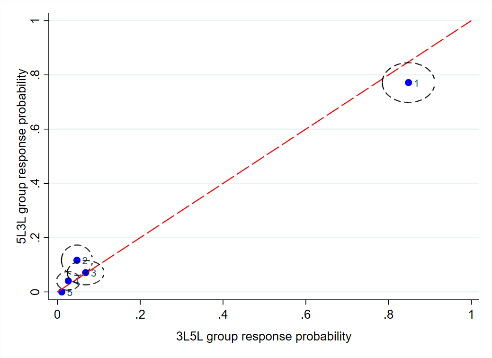 |
| 1. Self-care: subset with no LSI | 1. Self-care: subset with LSI |
| 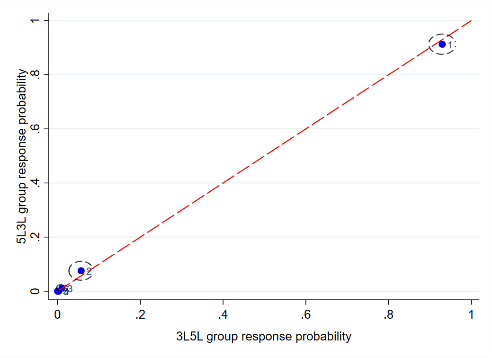 | 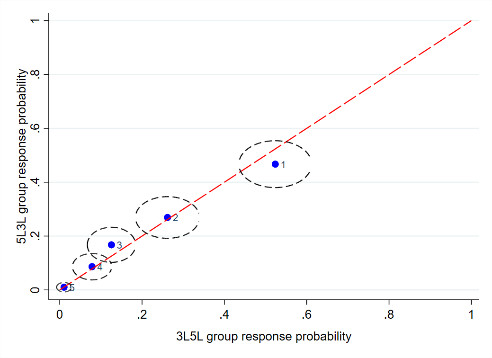 |
| 1. Activities: subset with no LSI | 1. Activities: subset with LSI |
| 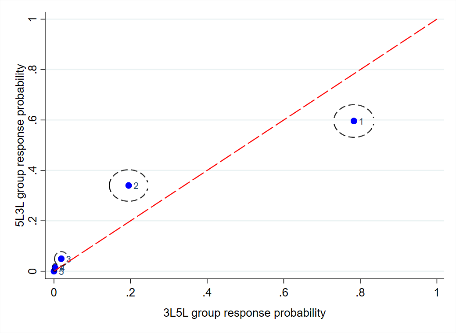 | 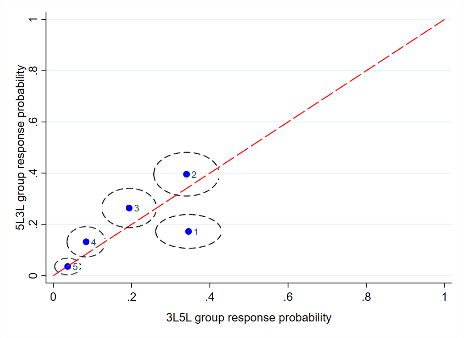 |
| 1. Pain: subset with no LSI | 1. Pain: subset with LSI |
| 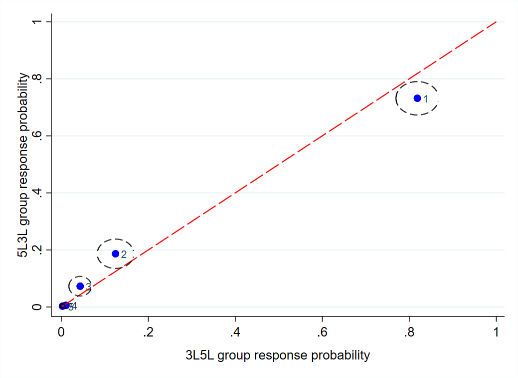 | 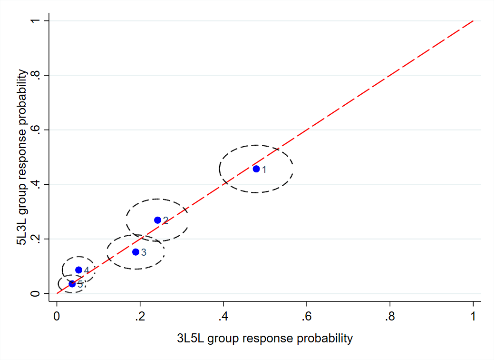 |
| 1. Anxiety: subset with no LSI | 1. Anxiety: subset with LSI |

| **Figure A4** 5L response probabilities with 95% confidence ellipses: *5L3L* vs*.5Lonly*, subsets of respondents without and with long-standing illness or disability (LSI) | |
| --- | --- |
| 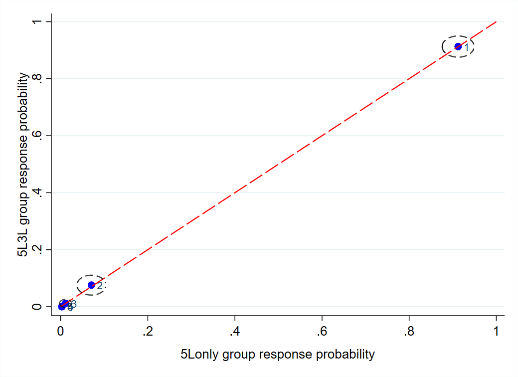 | 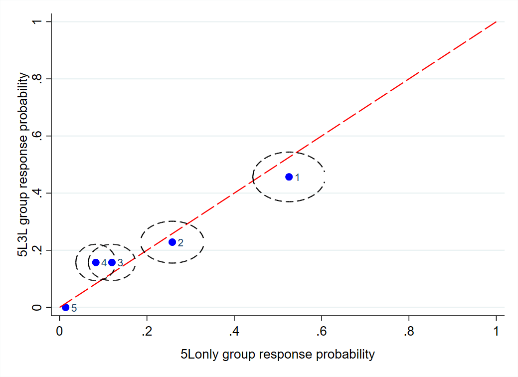 |
| 1. Mobility: subset with no LSI | 1. Mobility: subset with LSI |
| 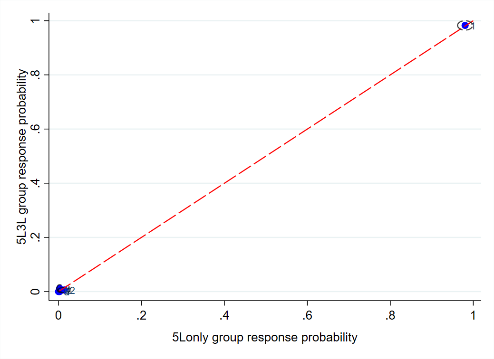 | 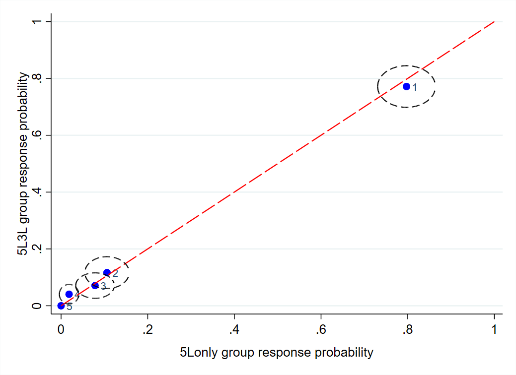 |
| 1. Self-care: subset with no LSI | 1. Self-care: subset with LSI |
| 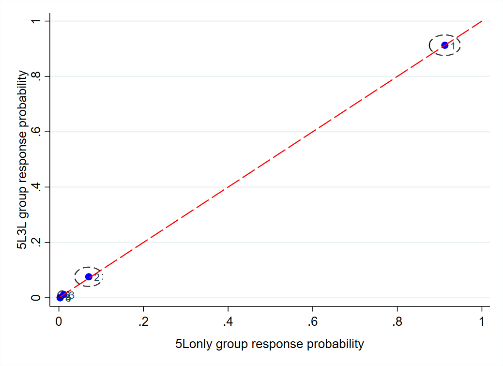 | 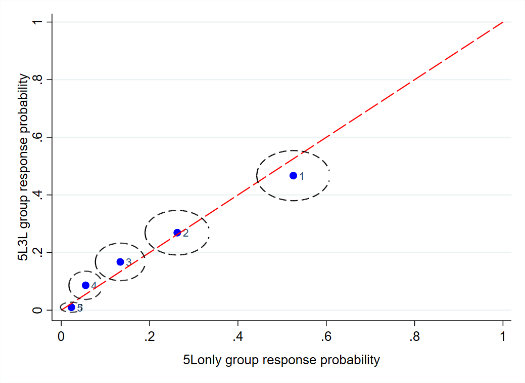 |
| 1. Activities: subset with no LSI | 1. Activities: subset with LSI |
| 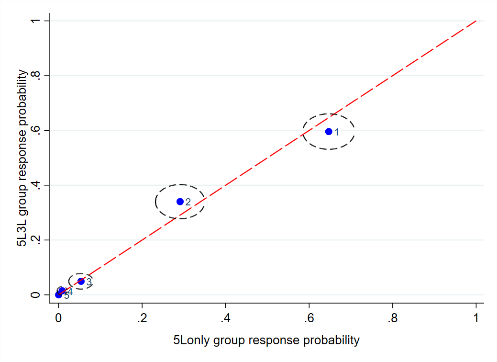 | 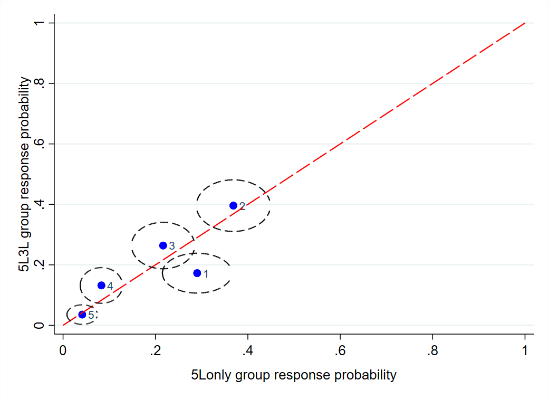 |
| 1. Pain: subset with no LSI | 1. Pain: subset with LSI |
| 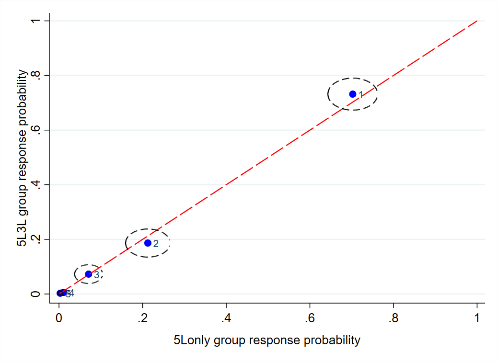 | 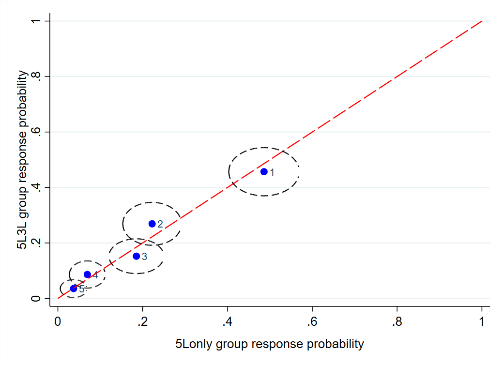 |
| 1. Anxiety: subset with no LSI | 1. Anxiety: subset with LSI |
| **Figure A5** 5L response probabilities with 95% confidence ellipses:  *3L5L* vs*.* *5Lonly*, subsets of respondents without and with long-standing illness or disability (LSI) | |
| 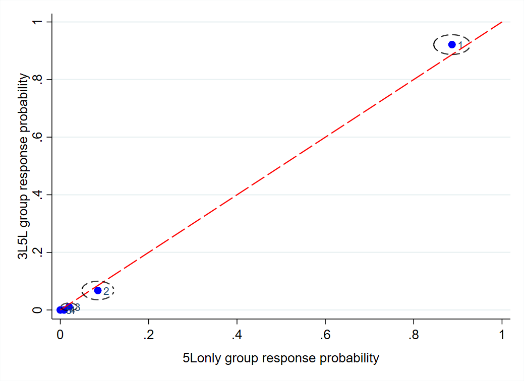 | 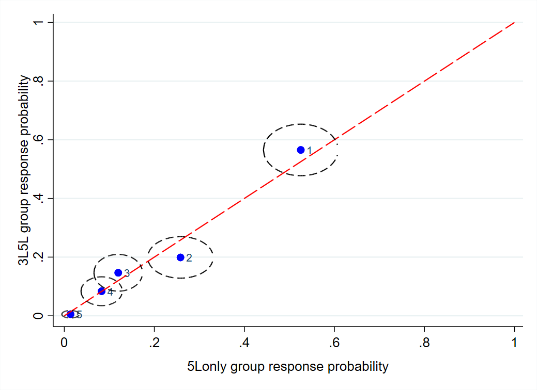 |
| 1. Mobility: subset with no LSI | 1. Mobility: subset with LSI |
| 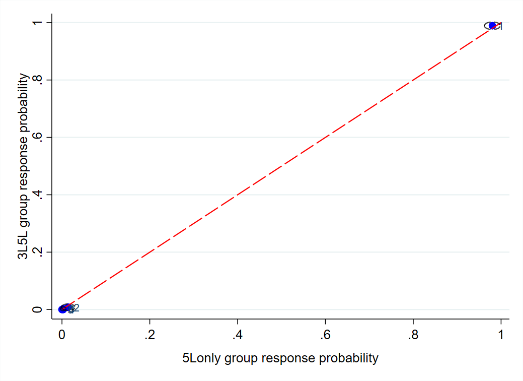 | 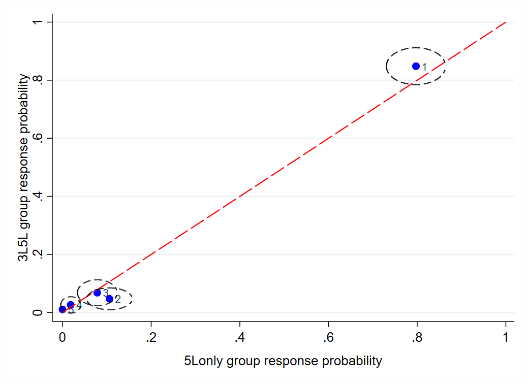 |
| 1. Self-care: subset with no LSI | 1. Self-care: subset with LSI |
| 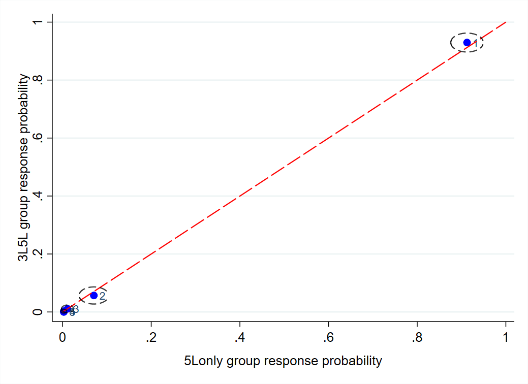 | 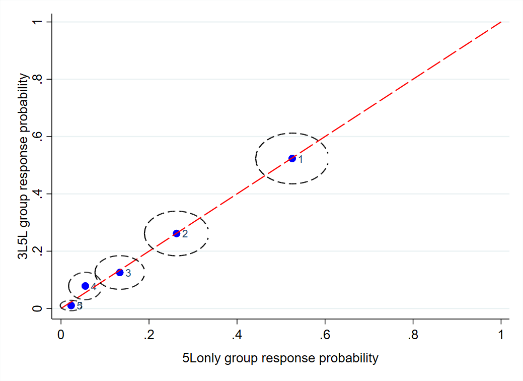 |
| 1. Activities: subset with no LSI | 1. Activities: subset with LSI |
| 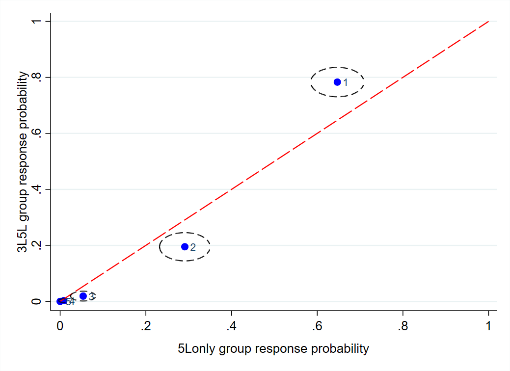 | 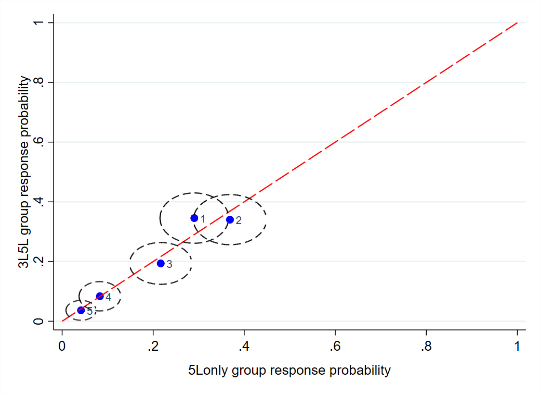 |
| 1. Pain: subset with no LSI | 1. Pain: subset with LSI |
| 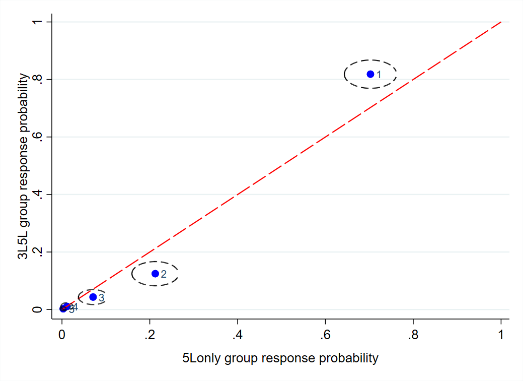 | 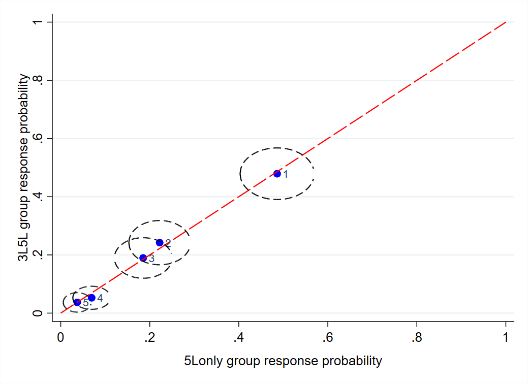 |
| 1. Anxiety: subset with no LSI | 1. Anxiety: subset with LSI |

| **Figure A6** 3L response probabilities with 95% confidence ellipses: *5L3L* vs. *3L5L*, subsets of respondents without and with long-standing illness or disability (LSI) | |
| --- | --- |
| 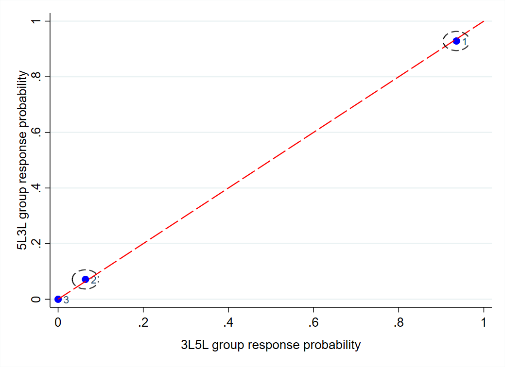 | 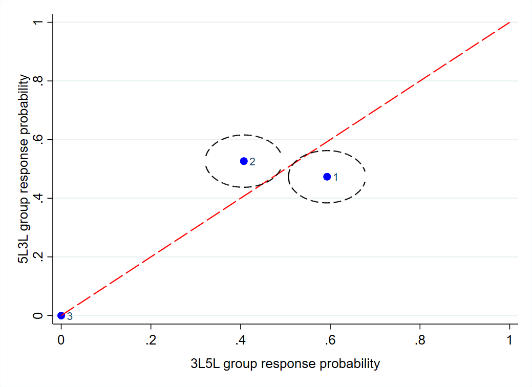 |
| 1. Mobility: subset with no LSI | 1. Mobility: subset with LSI |
| 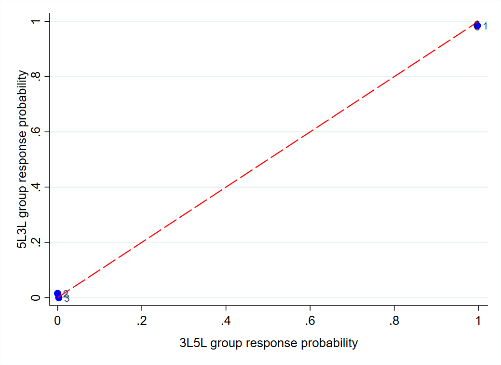 | 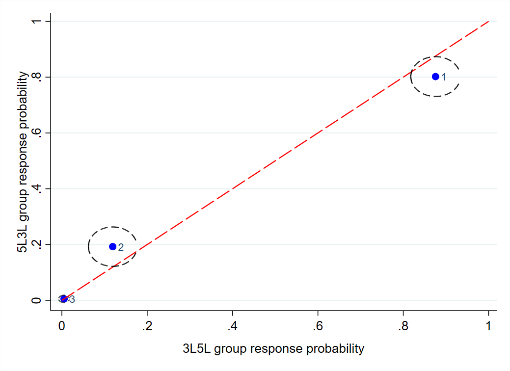 |
| 1. Self-care: subset with no LSI | 1. Self-care: subset with LSI |
| 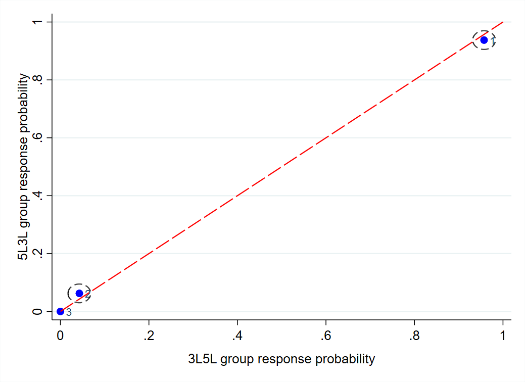 | 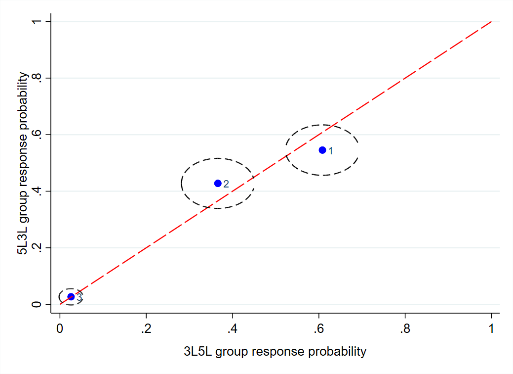 |
| 1. Activities: subset with no LSI | 1. Activities: subset with LSI |
| 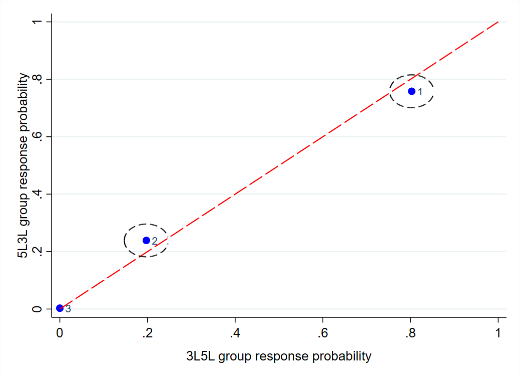 | 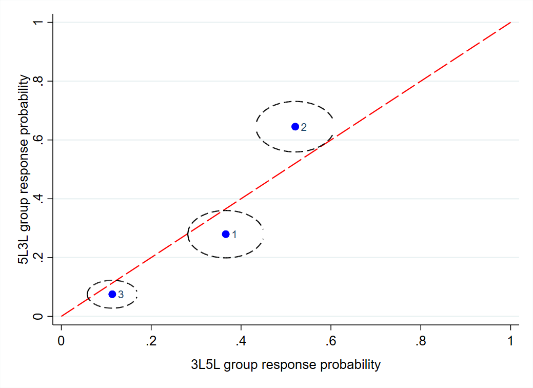 |
| 1. Pain: subset with no LSI | 1. Pain: subset with LSI |
| 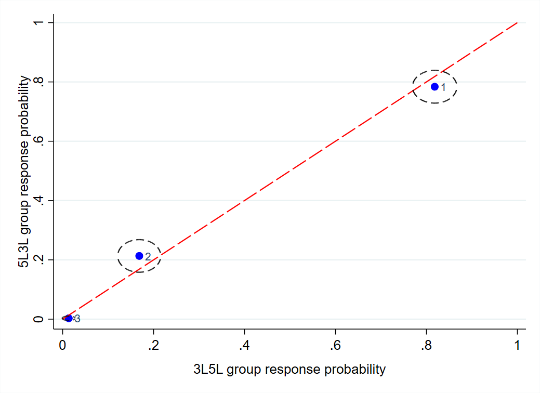 | 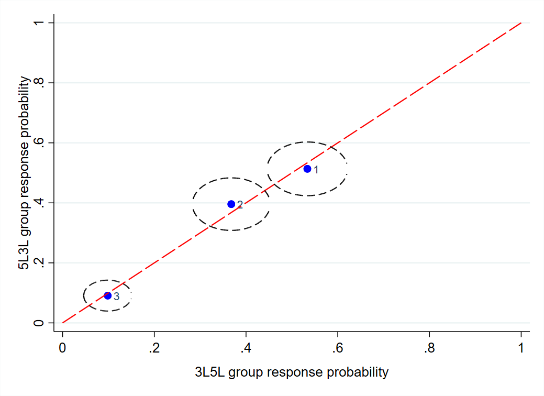 |
| 1. Anxiety: subset with no LSI | 1. Anxiety: subset with LSI |

**Figure A7** CASI/CAWI screens of pain dimension of the 3L and 5L.

| 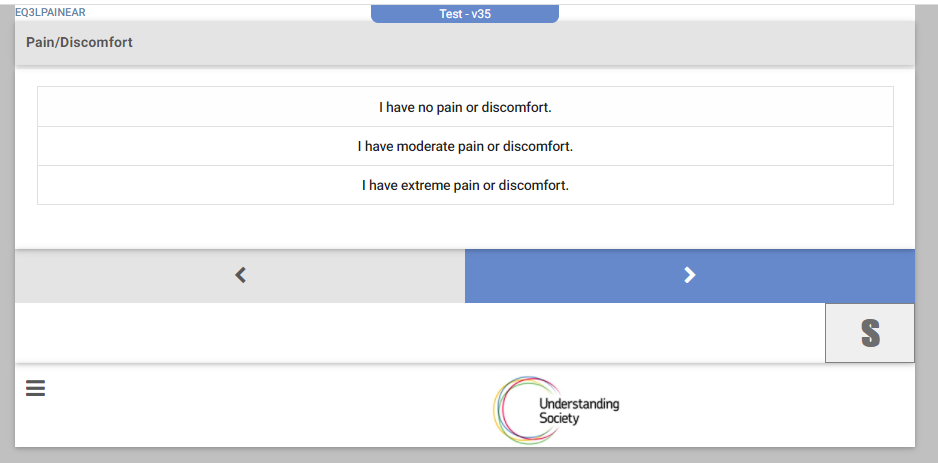   1. 3L |
| --- |
| 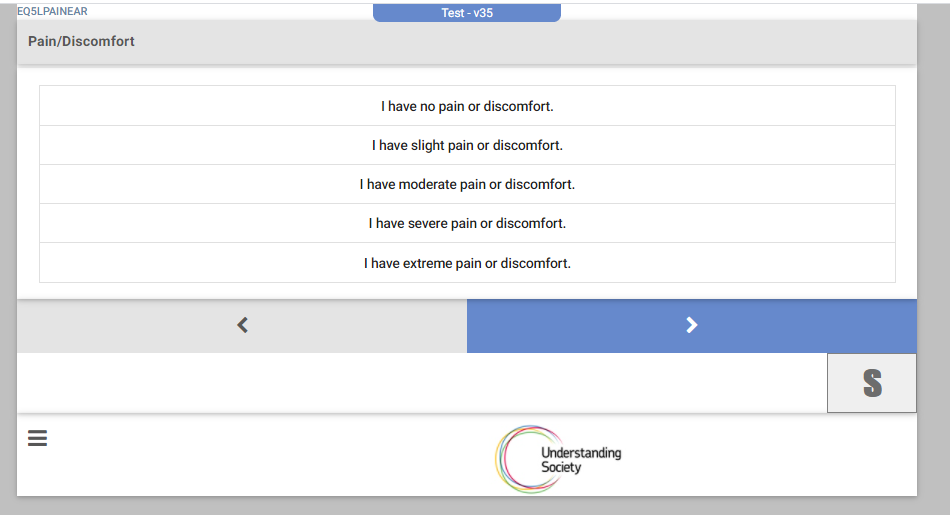   1. 5L |
